# β-hydroxybutyrate and its metabolic effects on age-associated pathology

**DOI:** 10.1038/s12276-020-0415-z

**Published:** 2020-04-08

**Authors:** Young-Min Han, Tharmarajan Ramprasath, Ming-Hui Zou

**Affiliations:** Center for Molecular and Translational Medicine, Georgia State University, Atlanta, GA USA

**Keywords:** Translational research, Mechanisms of disease

## Abstract

Aging is a universal process that renders individuals vulnerable to many diseases. Although this process is irreversible, dietary modulation and caloric restriction are often considered to have antiaging effects. Dietary modulation can increase and maintain circulating ketone bodies, especially β-hydroxybutyrate (β-HB), which is one of the most abundant ketone bodies in human circulation. Increased β-HB has been reported to prevent or improve the symptoms of various age-associated diseases. Indeed, numerous studies have reported that a ketogenic diet or ketone ester administration alleviates symptoms of neurodegenerative diseases, cardiovascular diseases, and cancers. Considering the potential of β-HB and the intriguing data emerging from in vivo and in vitro experiments as well as clinical trials, this therapeutic area is worthy of attention. In this review, we highlight studies that focus on the identified targets of β-HB and the cellular signals regulated by β-HB with respect to alleviation of age-associated ailments.

## Age-associated disease: Possible metabolic therapy

Boosting levels of a byproduct of fatty acid breakdown may help alleviate the symptoms of age-associated health conditions. When the body is low on glucose, it breaks down fatty acids for energy, generating byproduct metabolites called ketones. The ketone β-hydroxybutyrate (β-HB) regulates cellular signaling and gene and protein expression. There are indications that ketogenic diets or ketone administration, which increase β-BH may prevent ageing-associated progression of illnesses like cardiovascular and neurodegenerative diseases and cancer. Young-min Han and co-workers at Georgia State University in Atlanta, USA, reviewed current understanding of β-BH and its molecular targets. β-BH is a potent metabolite small enough to filter through cell membranes and circulate throughout the body, including the brain, influencing signaling pathways. Further investigations into associated molecular mechanisms will verify the metabolite’s potential as a therapeutic agent.

## Introduction

**Fig. 1:**
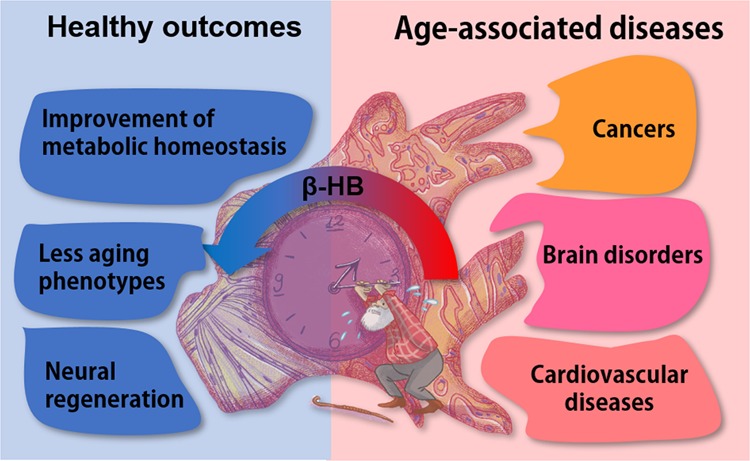
Alleviation of age-associated disease symptoms and improvement of health outcomes by increased β-HB. Aging is the leading risk factor for the development of various cancers, neurodegenerative diseases, and cardiovascular disease. Dietary manipulation, such as calorie restriction or a ketogenic diet, alleviates these age-associated diseases through upregulation of circulating β-HB. The increase in β-HB, as depicted by the redshift of the arrow in the figure, improves metabolic complications caused by insulin resistance, reduces cellular aging phenotypes, including senescence and inflammation, and regenerates sciatic nerves.

Aging, along with its associated ailments, including cardiovascular disease, cancer, arthritis, dementia, cataracts, osteoporosis, diabetes, hypertension, and Alzheimer’s disease, is a primary health concern. Accumulating data demonstrate that a ketogenic diet elevates the levels of β-hydroxybutyrate (β-HB), improving many age-related diseases (Fig. 1). Indeed, β-HB appears to act as a regulator of cellular signaling via numerous pathways in various cellular organelles in a manner that is independent of nicotinamide adenine dinucleotide (NAD) levels. Reports have verified that β-HB controls many cellular signals via its function as a ligand, regulates gene expression, inhibits or activates protein functions, and plays a role in neuronal functions. Thus, identification of the molecular targets of β-HB will provide a better understanding of how calorie restriction or a ketogenic diet improves age-related disease symptoms. Stemming from the current evidence, investigation of the detailed molecular capacity of β-HB will provide new opportunities for its application as a therapeutic target for the treatment or prevention of human diseases. Based on the potential of β-HB, this review describes the direct and indirect effects of β-HB on the regulation of signals involved in inflammation, senescence, and apoptosis. This review also describes the effect of ketogenic diets and/or ketone ester (KE) treatment as well as the molecular mechanisms and targets of these interventions on aging-related diseases, such as cancer, cardiovascular disease, and neurodegenerative diseases.

## Aging epidemics and the global public health emergency

A worldwide increase in the elderly population caused by declining fertility and increased life expectancy is thought to represent one of the most significant global health emergencies of the 21st century. Geriatric syndromes that affect this aging population are characterized by the emergence of several complex health states that render individuals vulnerable to many disease conditions. The number of people aged 65 years or older is projected to increase from an estimated 524 million in 2010 to nearly 1.5 billion in 2050, with the majority of the increase occurring in developing countries. While the invention of modern therapies extends life expectancy, these advancements also increase aging-based noncommunicable diseases that impose a considerable burden on the global population^1,2^. Reducing severe disability from disease is key to minimizing health and social costs as the population increases.

## Feeding-fasting effects on aging

At all stages of life, nutrient requirements are essential; however, overnutrition is strongly associated with serious health conditions and an enhanced aging process^3^. Various species of animals have developed diverse nutritional and metabolic mechanisms to adapt to sudden nutritional changes^4^. In particular, these diverse metabolic control mechanisms provide a resilient or flexible adaptation to stress from food insecurity as well as overeating^5^. Food availability has a very close and direct effect on health. Specifically, calorie restriction or intermittent fasting is currently the most effective dietary intervention to prolong a healthy lifespan^6^ and prevent cancer development^7^. Moreover, studies have demonstrated that caloric restriction improves memory in elderly humans^8^.

## β-HB levels contribute to antiaging phenotypes

**Fig. 2:**
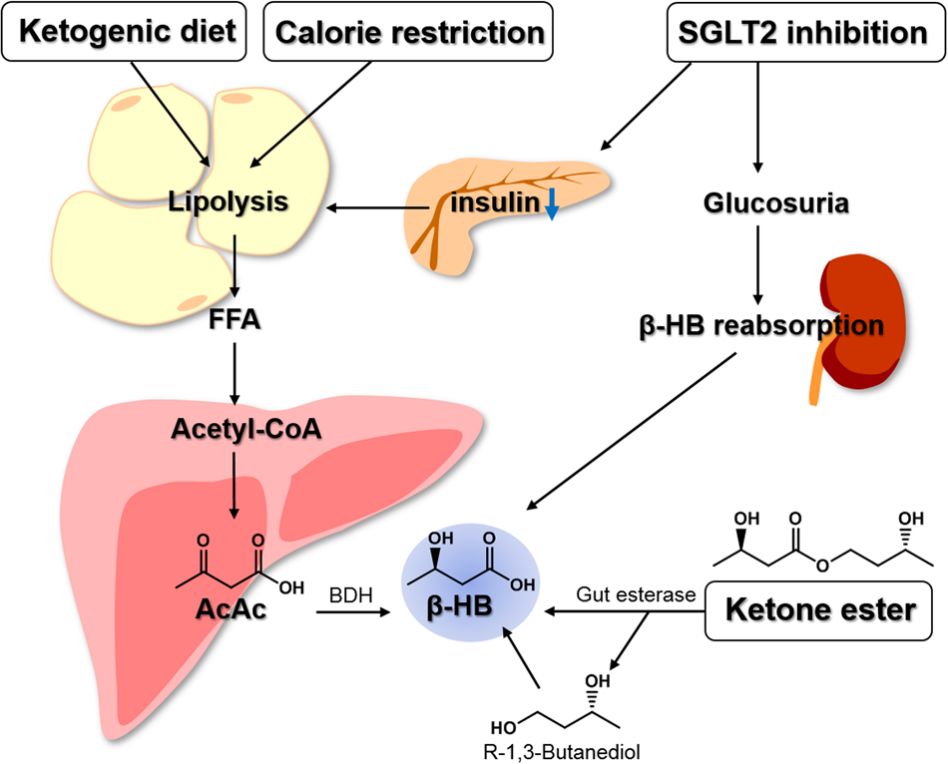
Dietary manipulation or supplementation to induce β-HB elevation. There are multiple ways to increase β-HB in circulation. The β-HB level is controlled by lipolysis in adipose tissue. A ketogenic diet and calorie restriction are the most well-known dietary manipulations used to stimulate lipolysis. Lipolysis-induced FFA is converted to β-HB through β-oxidation in the liver. SGLT2 inhibition also elevates β-HB levels by shifting substrate utilization from carbohydrate to ketone bodies through lipolysis or glucosuria. KEs have recently been developed as a commercially available β-HB supplement.

Approaches to increase circulating β-HB by dietary manipulation or ingestion of supplements have been examined via four different routes: a ketogenic diet, calorie restriction, KE administration, and sodium-glucose transport protein 2 (SGLT2) inhibition (Fig. 2). The ketogenic diet, calorie restriction, and SGLT2 inhibition induce ketogenesis in the liver through lipolysis. In particular, SGLT2 inhibition decreases insulin secretion from β cells, resulting in lipolysis in adipose tissues, regulation of ketone body reabsorption in the kidney, and increased β-HB^9^. KEs are hydrolyzed by nonspecific gut esterases in the small intestine and liberate β-HB and (R)-1,3-butanediol, thus increasing the level of β-HB in the circulation^10^.

Since ketone body metabolism is highly activated during calorie restriction or in glucose-deprived metabolic conditions, ketone bodies are considered crucial metabolites for calorie restriction-induced antiaging phenotypes^11^. Therefore, in-depth research is required to establish the effects of ketone body-associated therapeutic intervention on aging and age-related diseases. Physiologically produced ketones travel to peripheral tissues and are used as an energy source as an alternative to glucose. In particular, β-HB is the most abundant ketone body absorbed as an alternative energy source by peripheral tissues, such as the muscles, heart, and brain, in various animal species. β-HB is derived in the liver from the β-oxidation of free fatty acids (FFAs) that are produced via lipolysis of adipose tissues during periods of glucose deficiency. Circulating β-HB is converted to acetyl-CoA via the ketosis pathway in the muscles, heart, and brain, and this intermediate is then further used to generate adenosine triphosphate (ATP) via the citric acid cycle^12^. Although numerous studies have been conducted on the function of β-HB as an alternative energy source, the role of β-HB as a signaling metabolite during some pathophysiological conditions remains an unclear, albeit very interesting, topic.

## Benefits of β-HB on aging and associated diseases

### Aging

The effects of calorie restriction on prolonging life and delaying the onset of age-associated diseases in a variety of species, including rats, mice, fish, flies, worms, and yeast, have been demonstrated^8^. Nonetheless, the molecules and cell signals that underlie these effects remain elusive. Intensive efforts have revealed that a ketogenic diet contributes to a longer lifespan, similar to calorie restriction^13^. Circulating β-HB is the most significantly increased metabolite during caloric restriction and ketogenic diets, highlighting β-HB as an antiaging metabolite^11^.

Indeed, β-HB supplementation extends the lifespan of *C. elegans* by 20% through the DAF-16/FOXO and SKN-1/Nrf pathways and the regulation of aging and longevity^14^. In mammals, β-HB decreases the senescence-associated secretory phenotype (SASP) and the senescence of vascular cells^15^. Moreover, the ketogenic diet significantly extended the median lifespan of mice and resulted in the preservation of the physical function of aged mice^13^. Furthermore, a cyclic ketogenic diet was reported to reduce midlife mortality and improve memory performance in aged mice^16^.

Age reprogramming and epigenetic rejuvenation contribute to an increase in lifespan and improved rejuvenation of age and age-associated hallmarks^17^. Regenerative medicine studies have emerged, paving the way for new therapeutic interventions for such age-associated diseases. Although many studies on the ketogenic diet and its therapeutic effects in regenerative medicine during aging and specifically for neurodegenerative diseases continue to be published, the molecular mechanism of ketone bodies has not yet been explored thoroughly. A ketogenic diet showed a neuroprotective effect on the central nervous system (CNS) via the regeneration of sciatic nerves^18^. A ketogenic diet also restored oligodendrocyte integrity and increased CNS myelination in a murine Pelizaeus-Merzbacher disease model^19^. Furthermore, exogenous β-HB improved stem cell homeostasis and intestinal stem cell function through activation of Notch signaling, which is a key signaling axis for tissue regeneration^20^. Thus, β-HB and ketogenic diets can be considered important mediators with regenerative potential that also have the capacity to retard aging-associated phenotypes.

### Cancers

Aging is the most significant risk factor in the development of cancer, which is a leading cause of human mortality^21^. Cancer cells have significant alterations in metabolism, resulting in increased levels of mitochondrial-derived reactive oxygen species (ROS), such as O_2_^−^ and H_2_O_2_. Cancer cells prefer to switch to aerobic glycolysis, known as the Warburg effect, to compensate for the mitochondrial dysfunction induced by increased ROS levels^22^. Thus, lowering glucose availability to cancer cells offers a therapeutic option. A recent study suggests that a ketogenic diet enhances the cancer cell therapeutic response through selective metabolic oxidative stress^23^. Other animal studies support that a ketogenic diet inhibits the progression of the primary tumor^24^ as well as systemic metastasis^25,26^. Chronic intake of a carbohydrate-rich western diet results in high insulin and insulin-like growth factor (IGF-1) levels, promoting tumor cell proliferation^27^. Furthermore, cancer cells have been reported to increase their dependence on glucose in the blood in response to the demand for rapid cell growth, and other studies have suggested that glucose may have a direct or indirect effect on the proliferation of tumor cells. Carbohydrate-restricted ketogenic diets enriched in fat have been repeatedly reported to suppress breast cancer^28^. Additionally, ketone bodies used as a fuel source were reported to suppress cancer cell proliferation. In particular, researchers pointed out a higher incidence of breast cancer among individuals with diabetes and obesity, confirming that a low-carbohydrate diet may limit tumor growth^29^. Thus, considering the impact of carbohydrates in promoting breast cancer, the ketogenic diet has the potential to control or reduce one’s risk of developing breast cancer. Other studies also reported that a ketogenic diet may be particularly useful in the treatment of brain cancer^30,31^, as patients with the most common and aggressive form of brain cancer, glioblastoma multiforme, showed significant improvement following the adoption of a ketogenic diet^32^. Although it may not significantly affect disease progression in advanced and terminal cancers, a ketogenic diet is safe and has the potential to improve the quality of life in cancer patients in combination with radiation or other verified anticancer therapies^33,34^. The above evidence suggests that these therapies should be further investigated to explore ketones as potential adjuvant therapeutics with minimal toxicity.

### Neurological disorders

As life expectancy increases for the population, many more elderly people suffer from neurological disorders, such as epilepsy and dementia^35^. Recent findings have shown that epilepsy patients have a higher risk of dementia, particularly Alzheimer’s disease^36^. Excessive brain activity in epilepsy patients causes seizures. Seizure medications are effective only for some patients with epilepsy, while others do not respond to the medications or experience side effects. According to many reports, a ketogenic diet with a high-fat, low-carbohydrate intake results in significant improvement in untreatable epileptic children. The ketogenic diet as a primary treatment reduced epileptic seizures by more than half and has thus been used worldwide for incurable pediatric epilepsy^37,38^. Furthermore, other reports have documented improvements in epilepsy as well as other neurological diseases in patients who follow a ketogenic diet^39,40^. Interestingly, ketones were reported to alter gut microbiota to prevent seizures and spontaneous tonic-clonic seizures via modulation of hippocampal GABA/glutamate ratios^41^.

Alzheimer’s disease is the most common age-associated neurodegenerative disease and requires an effective therapeutic strategy due to its increasing socioeconomic burden. Peripheral aging caused by inflammation, immune cell skewing, senescence, and infection further promotes the incidence and progression of Alzheimer’s disease^42^. This progressive disease is characterized by tangles in the brain and an accumulation of β-amyloid plaques, which are known markers of Alzheimer’s disease and are thought to impair memory. Animal studies have shown that β-HB can potentially reduce amyloid plaques, and thus, mechanisms to increase levels of β-HB in the blood via a ketogenic diet, supplementation with KEs, or medium-chain triglyceride (MCT) oil are potentially relevant to Alzheimer’s disease therapy. In addition, rising ketone levels as a result of dietary manipulation with KEs or MCT oil have been shown to improve some Alzheimer’s disease symptoms^43–47^.

To assess the effect of the ketogenic diet on motor performance, two transgenic mouse lines, APP/PS1 mice (model of amyloid deposition) and Tg4510 mice (model of tau deposition), were used^44^. Model mice fed with a ketogenic diet for three months exhibited significantly better performance on rotarod behavioral testing than those in the control group independent of genotype. The data demonstrate that ketogenic diets may play an important role in enhancing motor performance in mouse models^44^.

Parkinson’s disease is a neurodegenerative disorder characterized by the loss of nigrostriatal dopaminergic neurons accompanied by mitochondrial respiratory deficiency^48^. Ketogenic diets are being explored as potential complementary therapies for Parkinson’s disease due to their protective effects on the brain and nervous system, as described for Alzheimer’s disease and epilepsy. Ketogenic diets have been shown to protect neurons of the substantia nigra against 6-hydroxydopamine neurotoxicity in rat animal models^48^. 1-Methyl-4-phenyl-1,2,3,6-tetrahydropyridine (MPTP) is a neurotoxin that causes dopaminergic neurodegeneration and mitochondrial deficiency reminiscent of Parkinson’s disease. In an MPTP-induced murine Parkinson’s disease model, the dopamine neurodegeneration caused by MPTP was partially protected by an infusion of ketone bodies. Injection of β-HB into mice conferred partial protection against the dopamine neurodegeneration and motor deficiency induced by MPTP^49^. Thus, infusion of ketone bodies or adoption of a ketogenic diet protected against nerve damage and improved motor function in Parkinson’s animal models^48,49^, suggesting further exploration of the potential for such treatments for Parkinson’s disease patients.

### Cardiovascular disease

Obesity is associated with cardiovascular disease and leads to metabolic complications, such as insulin resistance^50^. Ketogenic diets have been reported to reduce weight more effectively than pure calorie restriction or a low-fat diet^51–53^. In addition to weight loss, ketogenic diets decrease the levels of triglycerides, LDL cholesterol, and blood glucose and increase the levels of HDL cholesterol^52,54^. Another benefit is that this type of diet helps the individual feel less hungry, and the inhibitory effects of ketosis can also help the individuals consume fewer calories^55^. Low-carbohydrate, ketogenic diets are often practiced to lose or maintain weight, but the metabolic effects of prolonged exposure to this type of diet remain controversial, as prolonged intake of a ketogenic diet reduces insulin sensitivity and impairs glucose tolerance^56^. These results can quickly reverse the effects of obesity. Therefore, an intermittent ketogenic diet is considered to be effective in reducing obesity.

Metabolic syndrome, including glucose intolerance and type 2 diabetes, is associated with aging^57^. A ketogenic diet has been shown to improve glycemic control and diabetic complications in patients with type 1 and type 2 diabetes. These patients were able to stop or reduce their diabetes medications by eating ketones, reducing weight, and reducing triglycerides and blood pressure^58–60^. In rats, maintenance of a ketogenic diet for 8 weeks decreased sensitivity to peripheral insulin and impaired glucose tolerance; however, a return to the normal chow diet after a ketogenic diet resulted in a dramatic reversal of these effects. Thus, long-term maintenance of a ketogenic diet negatively affects glucose homeostasis, but this effect can be rapidly reversed upon cessation of a ketogenic diet^56^. Thus, it can be postulated that direct consumption of ketone bodies, specifically β-HB, is a more efficient way to control metabolic syndrome.

Nonalcoholic fatty liver disease (NAFLD), which is also common in the elderly, is closely related to type 2 diabetes, metabolic syndrome, and obesity. Individuals with NAFLD have an excess of intrahepatic triglycerides. A ketogenic diet significantly reduced hepatic triglycerides in subjects with NAFLD compared to calorie restriction^61^, and another report demonstrated an improvement of NAFLD in patients who adopted a ketogenic diet^62,63^.

### Molecular targets of β-HB

**Fig. 3:**
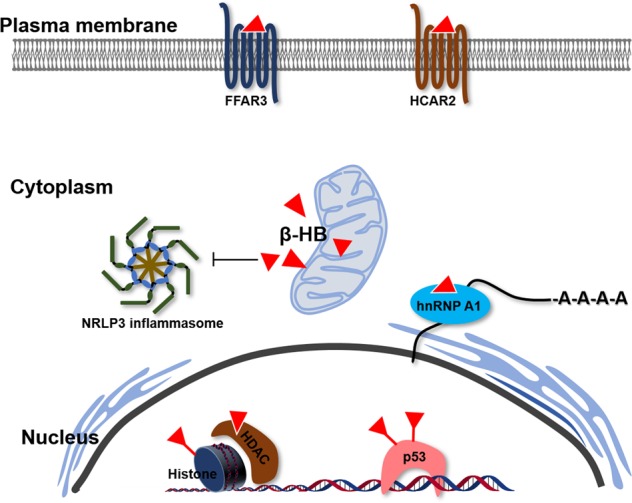
Inhibition, activation, and posttranslational modification by β-HB. β-HB directly or indirectly interacts with many cellular proteins in different organelles. β-HB acts as an agonist or antagonist to the two GPCRs FFAR3 and HCAR2 in the plasma membrane. β-HB directly binds to hnRNP A1 to regulate *Oct4* mRNA stability. β-HB suppresses inflammation through inhibition of NRLP3 inflammasome formation or its activity. Specifically, β-HB also regulates nuclear proteins. β-HB is an HDAC inhibitor that also regulates histones and p53 through β-hydroxybutyrylation.

The benefit of the ketogenic diet is now well understood by increasing scientific support. Being more than just a metabolite, β-HB has the capacity to trigger and control a variety of signaling events with implications for many metabolic diseases. However, it has a broad spectrum of targets at the molecular level, and the principal molecular targets are the NLRP3 inflammasome, RNA-binding proteins and G protein-coupled receptors (Fig. 3). In addition, β-HB has also been identified as an epigenetic modifier that can target DNA and histones. For example, β-HB is an endogenous inhibitor of many protein deacetylases (HDACs) and a β-hydroxybutyrylation modulator (Fig. 3), which is a new type of epigenetic regulatory mechanism. Thus, a clear understanding of the associations between β-HB metabolism and epigenetics would provide a way to develop new pharmacological interventions for the amelioration of a variety of pathological conditions.

### Histone deacetylases

Increasing β-HB by fasting or calorie restriction resulted in global histone acetylation in mice via inhibition of class I Histone deacetylases (HDACs) (HDAC1, 2, 3, and 8), which are also called sirtuins and comprise a family of proteins that regulate age-associated gene expression^64,65^. Inhibition of HDACs by β-HB upregulated the expression of the *Foxo3a* and *MT2* genes that encode oxidative stress resistance factors^65^. Additionally, β-HB prevented microglial process retraction and depressive-like behaviors by HDAC inhibition. β-HB-induced ramification and Akt activation in microglia abrogated HDAC activity, resulting in a further reduction in neuroinflammation in microglia^66^. Butyrate is a short-chain carboxylic acid produced in the gut. Recent studies reported that structurally and functionally similar butyrate inhibits HDAC more effectively than β-HB. In rodent models, supplementation with butyrate induces the expression of metabolically important genes that can improve insulin sensitivity linked to augmented energy expenditure^67^. This includes peroxisome proliferator-activated receptor gamma coactivator 1-alpha (PGC1-α), carnitine palmitoyltransferase 1B (CPT1b), mitochondrial sirtuins, superoxide dismutase 2 (SOD2), and catalase^68^.

### G protein-coupled receptors

β-HB has been reported to bind directly to hydroxycarboxylic acid receptor 2 (HCA2). HCA2 is a high-affinity G protein-coupled receptor (GPCR), which is a receptor for nicotinic acid with a half-maximal effective concentration (EC_50_) of 0.7 mM^69^. As GSK256073, a selective agonist of HCA2, lowers FFA levels via inhibition of lipolysis and glucose in type 2 diabetes^70^, β-HB may also reduce FFA and glucose levels via action as an HCA2 agonist to inhibit atherogenic activity.

HCA_2_ expression is not limited in adipocytes, but it is also expressed on neutrophils and resident macrophages as well as in the brain. In a stroke model, it was identified that HCA_2_ is required for the neuroprotective effect of β-HB and the ketogenic diet, as this effect is lost in HCA2^−/−^ mice. Further, ketogenic diet-induced activation of HCA2 is known to deliver neuroprotective signals through noninflammatory macrophage infiltration to the ischemic brain. β-HB, via its HCA2 agonist activity, induced a neuroprotective phenotype, as monocytes and macrophages rely on prostaglandin D2 (PGD2) production by cyclooxygenase 1 (COX1) and hematopoietic PGD2 synthase^71^. Mechanistically, PGD2 released by monocytes and macrophages mediates the neuroprotective effect of HCA2 by resolving inflammation and inhibiting IκB kinase (IKK) and NF-κB, which are key players in ischemic brain damage.

Another receptor of β-HB is FFA receptor 3 (FFAR3, GPR41), which controls body energy expenditure to maintain metabolic homeostasis. Activated by short-chain fatty acids (SCFAs) and β-HB that is produced by the liver upon starvation, this receptor inhibits N-type calcium channels and modulates the activity of sympathetic neurons through a signaling cascade that involves the β and γ subunits of its coupled G protein phospholipase C (PLC) and MAP kinases, such as ERK. Therefore, this factor may regulate energy expenditure through its effects on sympathetic nervous system control of heart rate. β-HB suppressed sympathetic nervous system activity by antagonizing FFAR3 during starvation or diabetic conditions^72^.

### RNA-binding proteins

β-HB directly binds several RNA-binding proteins, including heterogeneous nuclear ribonucleoprotein A1 (hnRNP A1), splicing factor proline and glutamine rich (SFPQ), and RNA-binding protein FUS/TLS. hnRNP A1 is a dominant binding partner of β-HB in vascular cells, such as endothelial and smooth muscle cells^15^. Upregulation of circulating β-HB delays the aging progress of mice by preventing vascular cell senescence. In addition, hnRNP A1 antagonized cellular senescence and SASP via stabilization of Oct4 and Sirt1 mRNAs^15,73^. In addition, a constitutive decrease in the levels and activities of hnRNP A1 or A2 in senescent fibroblasts is typically accompanied by an increase in the level of the p16 (INK4a) isoform, which is a primary aging marker^74^. Furthermore, SFPQ and FUS are also highly associated with age-associated neurodegenerative disease and amyotrophic lateral sclerosis^75,76^.

### NLRP3

Though multiple studies have indicated that calorie restriction or a ketogenic diet reduces oxidative stress and inflammation, the impact of a β-HB-mediated innate immune response remains unclear^77^. β-HB suppresses activation of the NOD-, LRR- and pyrin domain-containing protein 3 (NLRP3) inflammasome by preventing K^+^ efflux and reducing apoptosis-associated speck-like protein with caspase-recruitment domain (ASC) oligomerization and speck formation^78^. Interestingly, S-β-HB, a chiral enantiomer of β-HB, exhibits similar inhibitory capacity, but structurally related molecules, such as AcAc, butyrate, and acetate, do not inhibit NLRP3 activity^78^. Furthermore, NLRP3 inflammasome inhibition does not rely on uncoupling protein-2 (UCP2), sirtuin-2 (SIRT2), the GPCR FFAR3, or HCAR2, which have been described as target molecules of β-HB. These observations indicate that suppression of inflammation by calorie restriction or a ketogenic diet occurs through upregulation of β-HB targeting of the NRLP3 inflammasome^78^.

### β-hydroxybutyrylation

β-hydroxybutyrylation, a new type of histone modification, has been reported as an epigenetic regulatory mark enriched in active gene promoters^79^. Forty-four nonredundant β-hydroxybutyrylation sites on histone lysine residues were verified in human and mouse cells, offering insight into a new epigenetic regulatory mark that controls diverse gene expression in association with calorie restriction or a ketogenic diet^79^. This study also verified that β-hydroxybutyrylation is one of the posttranslational modifications of p53, which is one of the most widely studied tumor suppressors and is also highly associated with senescence and apoptosis. p53 activity has been shown to be finely tuned by various posttranslational modifications, including acetylation, methylation, phosphorylation, ubiquitination, sumoylation, and neddylation. β-hydroxybutyrylation is observed at acetylation sites at lysines 120, 319, and 370 of p53. Since β-hydroxybutyrylation competes with acetylation on p53, an increase in β-HB due to calorie restriction or a ketogenic diet would reduce p53 acetylation, affecting p53 activity^80^. Furthermore, β-hydroxybutyrylation was identified on histones, offering a new type of chromatin regulation as well^79^.

**Fig. 4:**
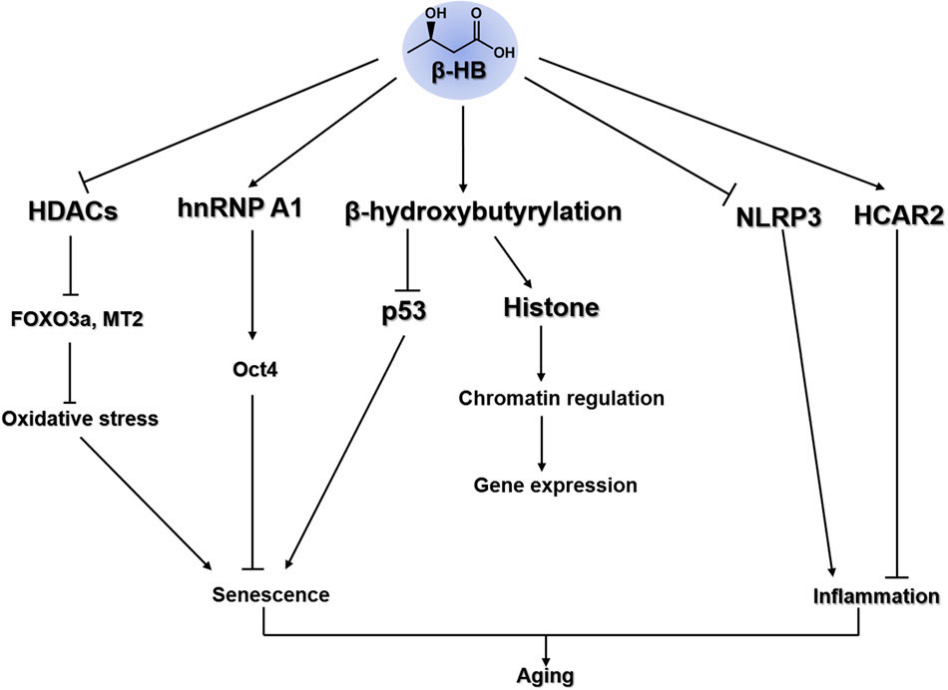
The overall molecular mechanisms underlying the β-HB-associated effects on aging. Target molecules and cellular signaling of β-HB are associated with the aging process, which is accelerated by senescence and inflammation. β-HB delays senescence via HDAC inhibition, hnRNP A1-mediated Oct4 expression, and β-hydroxybutyrylation on p53. Furthermore, β-HB suppresses inflammation by NLRP3 inhibition or HCAR2 activation and reduces the contribution to aging-associated diseases.

These results suggest that β-HB-mediated epigenetic and posttranslational modifications may play a critical role in regulating gene expression and signal transduction (Figs. 3, 4). Though the regulation of β-hydroxybutyrylation and the enzymes involved in this process are unclear, a recent report demonstrated that Sirt3 is a crucial enzyme involved in the de-β-hydroxybutyrylation of HDAC^81^. Further characterization of targets and regulators of β-hydroxybutyrylation could offer a novel approach to unveil the molecular mechanisms of β-HB in association with calorie restriction and a ketogenic diet.

### Perspectives

In addition to acting as an alternative energy source, β-HB is also a potent metabolite that regulates cellular signals by targeting diverse biomolecules. β-HB is a tiny molecule that can easily pass through cell membranes and circulate throughout the body in the blood vessels, even reaching the brain through the blood-brain barrier (BBB). This feature provides an advantage to β-HB over other existing drugs for the treatment of neurological diseases. In addition, β-HB also achieves passage across cell membranes and regulates proteins in various cellular organelles. β-HB affects signaling factors, including GPCRs in the cytoplasm, histones in the nucleus, and HDACs, hnRNP A1, and p53 in the cytoplasm. Numerous studies have reported that β-HB improves neurodegenerative diseases and aging-related cardiovascular disease. To date, the ketogenic diet has been used to relieve symptoms of diseases, such as cancer, metabolic syndrome, cardiovascular disease, and neurodegenerative diseases, as adjuvant therapeutic agents. Validation in additional in-depth studies will establish β-HB as a new therapeutic option for these conditions. Furthermore, the use of this factor as a therapy requires the optimization of the therapeutic dose of β-HB via pharmacokinetic studies in vivo. Since individual differences make it difficult to control the optimal circulating β-HB levels by calorie restriction or a ketogenic diet, it is necessary to develop adjustable treatment options, such as KE administration. As abrupt changes in circulating β-HB may disrupt energy homeostasis, the chiral enantiomer s-β-HB may offer a potential option for therapeutics, as this molecule cannot be used as an alternative energy metabolite. Furthermore, s-β-HB is not consumed by the physiological system, and the half-life of s-β-HB in circulation is longer than that of β-HB. As β-HB alleviates various age-associated disease symptoms and aging phenotypes via diverse and yet unknown molecular mechanisms, evaluation of β-HB and/or s-β-HB as a therapeutic agent is an important approach for the treatment of the aging population.
